# Applicability of the ankle-brachial-index measurement as screening device for high cardiovascular risk: an observational study

**DOI:** 10.1186/1471-2261-12-59

**Published:** 2012-07-30

**Authors:** Bianca LW Bendermacher, Joep AW Teijink, Edith M Willigendael, Marie-Louise Bartelink, Ron JG Peters, Machteld Langenberg, Harry R Büller, Martin H Prins

**Affiliations:** 1 Department of Vascular Surgery, Catharina Hospital, Eindhoven, The Netherlands; 2 Department of Epidemiology, Caphri Research School, Maastricht University, Maastricht, The Netherlands; 3 University Medical Centre Utrecht, Julius Centre for Health Sciences and Primary Health Care, Utrecht, The Netherlands; 4 Department of Cardiology, Academic Medical Centre, University of Amsterdam, Amsterdam, The Netherlands; 5 Gezondheidscentrum West, Den Bosch, The Netherlands; 6 Department of Vascular Medicine and General Practice, Academic Medical Centre, University of Amsterdam, Amsterdam, The Netherlands

## Abstract

**Background:**

Screening with ankle-brachial index (ABI) measurement could be clinically relevant to avoid cardiovascular events in subjects with asymptomatic atherosclerosis. To assess the practical impact of guidelines regarding the use of ABI as a screening tool in general practice, the corresponding number needed to screen, including the required time investment, and the feasibility of ABI performance, was assessed.

**Methods:**

An observational study was performed in the setting of 955 general practices in the Netherlands. Overall, 13,038 subjects of ≥55 years presenting with symptoms of intermittent claudication and/or presenting with ≥ one vascular risk factor were included. Several guidelines recommend the ABI as an additional measurement in selected populations for risk assessment for cardiovascular morbidity.

**Results:**

Screening of the overall population of ≥50 years results in ≈862 subjects per general practice who should be screened, resulting in a time-requirement of approximately 6 weeks of full time work. Using an existing clinical prediction model, 247 patients per general practice should be screened for PAD by ABI measurement.

**Conclusion:**

Screening the entire population of ≥50 years will in our opinion not be feasible in general practice. A more rationale and efficient approach might be screening of subsets of the population of ≥55 years based on a clinical prediction model.

## Background

Peripheral arterial disease (PAD) is a common disease, with a prevalence that increases with age and with the presence of vascular risk factors. The ankle-brachial-index (ABI) is a simple, inexpensive diagnostic test for PAD. Among well-trained operators, reproducibility is excellent, and the validity of the test for a stenosis above 50% in the arteries of the leg is high, with a sensitivity and specificity of approximately 80% and 96% respectively [1]. The ABI measurement as a diagnostic tool is therefore a very useful non-invasive tool for identifying symptomatic patients with atherosclerosis in primary care. An ABI below 0.9 is associated with an important increased risk of cardiovascular morbidity and mortality with a positive predictive value of 17.6% for a future cardiovascular event [2], and a relative risk of 3.34 for cardiovascular and cerebrovascular mortality [3]. It is well known that in symptomatic patients with PAD secondary prevention of a cardiovascular event by risk factor management and antiplatelet therapy is effective. However, subjects with asymptomatic atherosclerosis may well benefit from the same preventive measures, since the cardiovascular morbidity rate for asymptomatic PAD subjects was estimated at 76.8 per 1000 person-years at risk, compared with 13.6 for the non-PAD population [4]. Furthermore, much higher all-cause and cardiovascular mortality rates were observed in asymptomatic PAD subjects (42.8 and 35.8 per 1000 person-years, respectively) compared with non-PAD subjects (10.9 and 2.4 per 1000 person-years, respectively) [4]. Therefore, screening of asymptomatic subjects at risk is likely to be clinically useful.

The advice to perform a risk assessment whether to initiate a primary prevention strategy in all asymptomatic subjects is consistent with current international clinical practice guidelines, including the TASC II guideline [5] and the European Guideline [6]. In earlier guidelines the initial risk assessment was recommended to be performed using a multifactorial statistical model, such as the Framingham Risk Score [7,8], to maximize the benefit-cost ratio of primary prevention treatment [9]. However, it is shown that a low ABI doubled the 10-year risk of total mortality, cardiovascular mortality, and major coronary event rate compared with the overall rate across all Framingham risk categories [10]. The ABI is recommended as additional measurement in selected populations, especially in people aged 50 years and older or those who appear to be at risk and therefore refining the risk prediction with improvement of the benefit-cost ratio. Besides, it is shown that the ABI provided independent risk information compared with the Framingham Risk Score [10].

Primary care providers are best positioned to determine the at-risk population in the general population and to initiate educational, lifestyle, and cardiovascular risk reduction therapies [11]. However, physicians have not readily adopted the screening of asymptomatic PAD in their general practice [12], and studies on the feasibility of ABI testing for the assessment of overall cardiovascular risk are lacking. Furthermore, to our knowledge, no guideline provides information on the necessity of repeating the ABI measurement, let alone its timing in subjects with a normal ABI (≥0.9) at first screening. Only for patients with diabetes a recommendation is given to repeat the ABI measurement every 5 years if the initial test is normal [13]. One could imagine that in subjects between 50 and 60 years of age with a normal initial ABI, the ABI can decrease over time due to the relatively slow progression of the atherosclerotic process without causing complaints. It is a possibility that subjects with this decrease in ABI should be a prime target for aggressive risk factor management. Hence, it might be preferable to repeat the ABI measurement for screening of the presence of PAD, and consequently generalized atherosclerosis, in subjects with a normal initial ABI.

To assess the practical impact of the guidelines regarding the use of the ABI as a screening tool for diagnosing PAD in asymptomatic subjects, the number needed to screen was explored. The impact of this number needed to screen on the required time investment by general practitioners in the Netherlands was studied to be able to explore the feasibility of the ABI in general practice.

## Methods

Contemporary guidelines regarding the advice to perform an initial risk assessment for cardiovascular morbidity in clinical practice, were systematically searched for indications that an ABI measurement should be performed. For the guideline search MEDLINE and websites of guideline development organizations were used.

To assess the number needed to screen, a composition of the general population was made using the census of the Dutch population, provided by the Central Office of Statistics of the Netherlands, and studies reporting the prevalence of vascular risk factors in open study populations, taking into account the age band distribution. There were 6,087,661 people of 50 years and older with an overall population size of 16,754,989, corresponding with a population of 36.7% of 50 years and older.

To investigate an alternative strategy to restrict the ABI measurement to patients who are at a high risk, the PREVALENT clinical prediction model was used (Tables 1 and 2) [14]. This model has been developed by performing an ABI in 7.454 consecutive patients of 55 years of age and older, presenting with at least one vascular risk factor (e.g. smoking, hypertension, diabetes mellitus, and hypercholesterolemia), without symptoms of PAD. Based on the prevalence of PAD related to risk factors, this PREVALENT clinical prediction model was developed. Taking a score limit of 7 or more risk factor points, resulting in a likelihood of approximately 20% or higher for an ABI below 0.9, the following populations should be screened. First, all current smokers of 55 years or older should be screened by ABI. With a prevalence of current smoking of approximately 20% in the population of 55 years and older, approximately 139 patients need to be screened in a general practice. A second population that should be screened for asymptomatic PAD are the subjects of 65 years or older with a history of smoking of 10 or more packyears and non-adequately treated hypertension, defined as a systolic blood pressure ≥140 mmHg and/or diastolic blood pressure ≥90 mmHg. Finally, the population of 75 years or older with non-adequately treated hypertension should be screened according to the PREVALENT clinical prediction model. Based on this model, only 48% of the asymptomatic population of 55 years and older will have a score of 7 or more, and are needed to be screened. Eventually, in 846 people an ABI below 0.9 will be measured using this clinical prediction model, compared to 1,299 in the overall screened population of 55 years and older.

**Table 1:** Clinical prediction model

**Risk factor points**	**Age**	**Smoking behaviour**	**Hypertension**
0	55 – 59 years	Never smoked	No hypertension
+ 1	60 – 64 years		Hypertension, adequately treated
+ 2	65 – 69 years	Ever smoked	
+ 3	70 – 74 years		Hypertension, not adequately treated
+ 4	75 – 79 years		
+ 5	80 – 84 years		
+ 6	≥ 85 years		
+ 7		Current smokers	

**Table 2:** Prevalence of PAD according to the clinical prediction model in asymptomatic subjects

**Score**	**Prevalence (n)**	ABI* < 0.9 (n -%)
0 – 3	1202	84 (7.0)
4	706	84 (11.9)
5	924	134 (14.5)
6	865	151 (17.5)
7	920	178 (19.3)
8	722	170(23.5)
9	448	116 (25.9)
10	470	114 (24.3)
11	331	83 (25.1)
12	241	75 (31.1)
≥ 13	271	110 (40.6)

*ABI: ankle-brachial index.

Furthermore, the time-investment of the ABI measurement was studied. Patients of 55 years and older with symptoms of intermittent claudication according to the general practitioner (without confirmation by ABI) and/or presenting with at least one vascular risk factor, were asked to participate in this observational study. There were no exclusion criteria. Informed consent was obtained from eligible patients. For the measurement of ABI, first the systolic brachial blood pressure was performed by auscultation at both arms, after which the systolic pressures of the dorsalis pedis and posterior tibial arteries were measured at malleolar level by an 8 MHz Doppler sound in both legs. The ABI was calculated for each leg by dividing the highest systolic ankle pressure by the highest brachial systolic pressure. The ABI was measured by the general practitioner or practice assistant. PAD was defined as a single ABI measurement of less than 0.9 in one or both legs.

After completing the case record form, the time required for an ABI measurement was reported for each patient. Furthermore, the general practitioner was asked if he had previous experience with performing the ABI measurement before participating in this study and who actually performed the ABI (e.g. general practitioner or practice assistant).

Finally, to explore the impact of the number needed to screen on the required time investment by general practitioners in the Netherlands, the number needed to screen was translated to general practice and related to the time requirement of an ABI measurement.

The study protocol was approved by the medical ethical committee of the Atrium Medical Centre Parkstad, Heerlen, the Netherlands.

## Results

### Contemporary guidelines

Overall, 6 international contemporary guidelines were included where an ABI measurement is advised to perform as initial risk assessment for cardiovascular morbidity [5,6,11,13,15,16]. Table 3 is showing an overview of these included guidelines with the recommended target population to perform an ABI measurement. The rationale for screening subjects with diabetes is that PAD is prevalent in patients with diabetes mellitus and is more commonly asymptomatic and more likely to lead to limb loss if a clinician waits until the onset of symptoms to identify disease [13]. It is suggested that the ABI has the potential to increase the sensitivity, specificity, and positive predictive values of cardiovascular risk. Although establishment of PAD diagnosis in individuals at-risk has the potential to alter the intensity of treatment goals [11], it should be noted that the impact of early PAD detection on either limb or cardiovascular ischemic event outcomes or on survival, has not yet been evaluated in prospective trials [11].

**Table 3:** Overview of included international guidelines with their recommended target population

**Guideline**	**Target population to perform an ABI of asymptomatic subjects**
ACCF/AHA 2011 [11]	· Age 65 years and older
	· Age 50 years and older with a history of smoking or diabetes
ACCF/AHA/ACR/SCAI/ SIR/SCM/SVN/SVS 2010 [15]	· Age 50–69 years with a history of smoking or diabetes
	· Age ≥ 70 years
TASC II 2006 [5]	· Age 50–69 years with cardiovascular risk factors
	· Age ≥ 70 years
	· Subjects with a 10-year risk of a cardiovascular event between 10-20% in whom further risk stratification is warranted
European guideline 2007 [6]	· Age ≥ 50 years
American Diabetes Association 2003 [13]	· Age > 50 years with diabetes
	· If normal, the test should be repeated every 5 years
Prevention conference V 2000 [16]	· Age ≥ 50 years

### Population composition

The overall population of 55 years of age and older in the Netherlands consists of approximately 4.9 million people (29.6%), with approximately 2.1 million male subjects [17].

### Smoking

It is reported that in 2010 of the people between 50 to 55 years, 55 to 65 years, 65 years to 75 years and 75 years and over respectively 31%, 25%, 18%, and 10% were current smokers [17]. Distribution to the general population in respective, taking the age band into account, this corresponds to an overall percentage of approximately 22.2% current smokers in the population of 50 years and older. Furthermore, it is described that over 40% of the women and over 65% of men aged above 50 years were former smokers [18].

In the population of 55 years and older, these percentages will be slightly less, due to the decreasing prevalence in older subjects, corresponding to approximately 20% current smokers.

### Hypertension

Due to the recent change in the cut off points for the definition of hypertension, there are no exact data available about the prevalence of hypertension, defined as a systolic blood pressure ≥140 mmHg and/or diastolic blood pressure ≥90 mmHg [19]. The ERGO study showed a prevalence of 27% in the subjects of 55 to 59 years of age [20]. A cross sectional survey consisting of 530 subjects of 55 years and older, found a prevalence of hypertension of 41.7%, defined as systolic blood pressure ≥140 mmHg and/or diastolic ≥90 mmHg [21]. Based on this, we estimated that there are currently approximately 2.04 million people of 55 years and older with hypertension in the Netherlands.

### Diabetes mellitus

Overall, there are approximately 696,150 patients with known diabetes mellitus in the Netherlands [17]. Approximately 89% is above 50 years of age, corresponding with approximately 620,000 subjects with diabetes (10.2%) [17]. The incidence of new diabetes is estimated to be approximately 58,090 subjects per year, with 82% older than 50 years of age [22].

### Hypercholesterolemia

No exact data about the prevalence of hypercholesterolemia are available due to the recent change in the cut off levels for the definition of hypercholesterolemia. Dutch data, using a definition of hypercholesterolemia of 6.5 mmol/l or higher and/or the use of lipid lowering medication, estimated the prevalence of hypercholesterolemia to be 33.9% in the population of 50 to 60 years of age [23].

A summary of prevalences of these common vascular risk factors are given in Table 4. Based on these independent prevalence’s of these risk factors, it can be calculated that less than 10% of subjects above 55 years are without any vascular risk factor (22.2% smokers, 27% subjects with hypertension, 10.2% subjects with diabetes mellitus, and 33.9% subjects with hypercholesterolemia). This low percentage is largely explained by current smoking behaviour and the presence of diabetes.

**Table 4:** Estimated prevalences* of vascular risk factors

**Vascular risk factor**	**Age ≥ 50 years**
Smoking	
Current smokers	≈ 22.2% (≈ 20% for age ≥ 55 years)
History of smoking	≈ 51%
Hypertension	≈ 41%
Diabetes Mellitus	≈ 10.2%
Hypercholesterolemia	≈ 34%

*Prevalences are based on the census of the Dutch population.

### Time-investment using the ABI

The study included 13,038 patients, performed by 955 general practices. Of the 513 general practitioners who included ≥ 20 patients, 101 (19.7%) recorded to have experience performing the ABI prior to participation in this study. The mean time necessary for ABI measurement in the overall population was 17 minutes (SD: 7.4). In 2,534 subjects (19.4%) ABI was measured by an experienced general practitioner or practice assistant. The influence of experience on the duration of ABI measurement is shown in Table 5.

**Table 5:** Influence of experience on measurement time of the ABI

**Included patients**	**Time ABI measurement**^**1**^**(min) Mean (SD)**			
	**Experienced**		**Non-experienced**	
	**GP**^**2**^	**PA**^**3**^	**GP**^**2**^	**PA**^**3**^
Patient 1	13.5 (5.9) (n = 47)	19.7 (9.5) (n = 78)	18.0 (8.1) (n = 73)	19.1 (8.1) (n = 206)
Patient 2	14.2 (5.9) (n = 47)	18.8 (8.7) (n = 76)	16.7 (6.9) (n = 71)	19.0 (7.3) (n = 201)
Patient 3	13.1 (5.4) (n = 43)	19.6 (10.4) (n = 78)	17.1 (7.5) (n = 71)	18.8 (7.3) (n = 205)
Patient 4	12.4 (4.7) (n = 45)	18.4 (7.8) (n = 77)	16.6 (7.5) (n = 70)	18.1 (7.1) (n = 205)
Patient 5	12.6 (5.8) (n = 47)	18.4 (6.9) (n = 76)	16.4 (9.6) (n = 71)	18.1 (6.9) (n = 202)
Patient 6 – 10	12.8 (5.7) (n = 228)	17.8 (7.4) (n = 367)	16.0 (7.8) (n = 354)	18.0 (7.5) (n = 993)
Patient 11 – 15	12.2 (5.2) (n = 211)	17.7 (7.3) (n = 355)	15.3 (7.5) (n = 341)	17.5 (7.2) (n = 947)
Patient ≥ 16	12.5 (5.4) (n = 200)	18.0 (7.5) (n = 347)	15.5 (7.2) (n = 335)	17.4 (6.9) (n = 936)

^1^Time ABI measurement included time needed to perform systolic pressures of the brachial artery at both arms and of the dorsalis pedis and posterior tibial arteries at both legs. ^2^GP: general practitioner; ^3^PA: practice assistent.

### Feasibility of the ABI as screening tool in general practice

Supposing that a general practice consists of approximately 2,350 patients, screening of the overall population of 50 years and older, according to the guidelines, corresponds with approximately 36.7% of the total population [17]. This results in a total of approximately 862 subjects per general practice who should be screened by ABI measurement.

**Figure 1:**
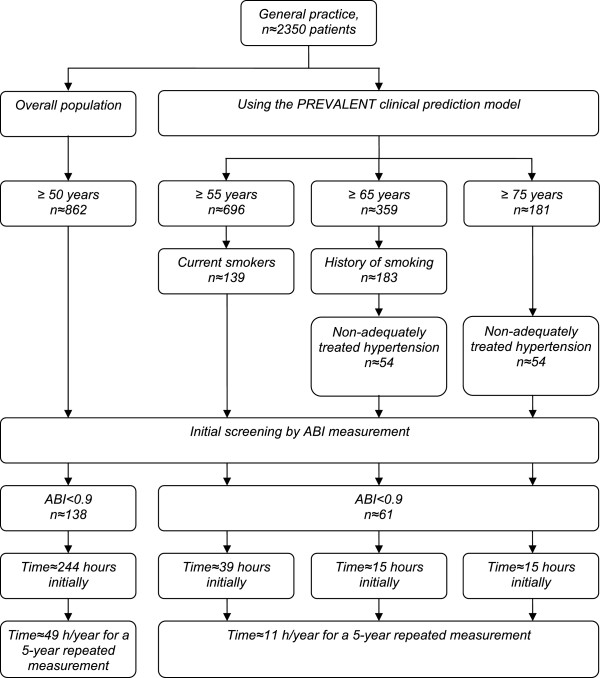
Feasibility of ABI screening.

Thus, a single screening of ABI in the overall population of 50 years and older would take an investment of approximately 6 weeks (244 hours) of full time work. Hence, a strategy of an ABI measurement in all subjects of 50 years and older to be performed every 5 years carries the burden of over one week full time work per year (Figure 1). Limitation of screening subjects of 50 years and older with at least one vascular risk factor only would not substantially reduce this workload due to the high prevalence of these vascular risk factors.

Using the PREVALENT clinical prediction model, first all current smokers of 55 years or older should be screened by ABI. With a prevalence of current smoking of approximately 20% in the population of 55 years and older, approximately 139 patients need to be screened in a general practice. The second population at risk are subjects of ≥65 years with inadequately treated hypertension, and a history of smoking behaviour. Since there are approximately 359 patients of ≥65 years, with approximately 29.6% inadequately treated hypertension, and approximately 51% former smokers, this results in approximately 54 subjects to screen for asymptomatic PAD in this subgroup. Assuming that in a general practice there are approximately 181 patients of 75 years and older, with approximately 29.6% inadequately treated hypertension, there are approximately 54 subjects to screen in this subgroup. Figure 1 shows the feasibility of the ABI screening using the clinical prediction model.

Overall, using the PREVALENT clinical prediction model, 247 patients per general practice should be screened for PAD by ABI measurement, corresponding to approximately 70 working hours for an initial screening measurement. Of the screened subjects, 60% to 80% will have a normal ABI (0.9 or higher). Screening these non-PAD subjects every five years, implies that in the following years approximately 150 to 200 subjects a repeated ABI measurement will have to be performed to diagnose asymptomatic PAD, excluding new subjects of 55 years of age. Hence, a strategy of an ABI measurement in these subjects to be performed every 5 years will only take approximately 11 working hours of full time work per year to diagnose asymptomatic PAD (Figure 1).

## Discussion

Following the recommendations of the current international guidelines regarding the use of an ABI measurement, will substantially increase the workload of general practitioners. The time requirement of 17 minutes per measurement is comparable with earlier results [24]. Although in the Netherlands the cost of measurement of the ABI in office practice is reimbursed by healthcare payers by an amount of 54.72 Euro for each ABI measurement, it is doubtful if the recommendations are feasible and can be followed in general practice, taking the high time pressure into account.

Based on the PREVALENT clinical prediction model, 52% of the subjects of 55 years and older will not be screened. Of these, approximately 12% will have an ABI below 0.9. However, of the screened population, the diagnosis PAD will be established by ABI measurement in 25%. Overall, approximately 65% of the asymptomatic patients with PAD will be found as a result of screening using clinical prediction model and ABI measurement.

### Clinical consequences of detecting a low ABI in asymptomatic subjects

Current U.S. national hypertension and lipid treatment guidelines include all patients with lower extremity PAD, regardless of symptom status, as a high-risk category [11]. In these guidelines, all patients should achieve risk reduction and specific treatment targets comparable to individuals with established coronary artery disease [25,26].

### Strengths and limitations of the study

This study is showing that screening the entire population of 50 years and older, as is advised in current international guidelines, will not be feasible in general practice, since the work involved is substantial. A more rationale approach might be the screening of subgroups of the population of 55 years and older based on PREVALENT clinical prediction model. The work load of screening can efficiently be reduced, while the majority of asymptomatic subjects with PAD will be detected.

The main limitation of the present study is that the clinical prediction model that is used in the calculations has not been validated yet. Furthermore, there are no exact data available of prevalences of vascular risk factors, making it difficult to do the calculations. Finally, no benefit-cost ratio analysis is performed, which might contribute even more to our statement that screening the entire population is not feasible in general practice.

## Conclusion

Screening for PAD by using ABI in the initial risk assessment is recommended as additional measurement in the population of 50 years and older. Screening the population of 50 years and older as prescribed by international guidelines, will not be feasible in general practice, since the work involved is substantial. A more rationale approach might be the screening of subgroups of the population of 55 years and older based on a clinical prediction model. Our calculations suggest that using the PREVALENT clinical risk score, the work load of screening can be reduced by 60% while the majority of asymptomatic patients with PAD (63%) will be detected. Ideally, cost effectiveness of screening with ABI measurement should be assessed in future studies.

## Competing interests

The authors declare that they have no competing interests.

## Authors’ contributions

BLWB, JAWT and MHP carried out the literature search, statistical analysis, and drafted the manuscript. EMW, MLB, RJGP, ML and HRB participated in the design of the study and coordination and helped to draft the manuscript. All authors read and approved the final manuscript.

## Pre-publication history

The pre-publication history for this paper can be accessed here:

http://www.biomedcentral.com/1471-2261/12/59/prepub
